# Activation Energies and Temperature Dependencies of the Rates of Crystallization and Melting of Polymers

**DOI:** 10.3390/polym12051070

**Published:** 2020-05-07

**Authors:** Sergey Vyazovkin

**Affiliations:** Department of Chemistry, University of Alabama at Birmingham, 901 S. 14th Street, Birmingham, AL 35294, USA; vyazovkin@uab.edu

**Keywords:** activation energy, Arrhenius equation, crystallization, differential scanning calorimetry (DSC), melting, multi-step kinetics

## Abstract

The objective of this review paper is to survey the phase transition kinetics with a focus on the temperature dependence of the rates of crystallization and melting, as well as on the activation energies of these processes obtained via the Arrhenius kinetic treatment, including the treatment by isoconversional methods. The literature is analyzed to track the development of the basic models and their underlying concepts. The review presents both theoretical and practical considerations regarding the kinetic analysis of crystallization and melting. Both processes are demonstrated to be kinetically complex, and this is revealed in the form of nonlinear Arrhenius plots and/or the variation of the activation energy with temperature. Principles which aid one to understand and interpret such results are discussed. An emphasis is also put on identifying proper computational methods and experimental data that can lead to meaningful kinetic interpretation.

## 1. Introduction

Evidently, it is a fact of nature that the rate of a broad variety of processes responds exponentially to temperature changes. For chemical reactions, this fact is reflected in a number of empirical equations [1], including the one proposed by Arrhenius [2]. Among those, the Arrhenius equation has thrived because it was backed up by the theories of the activated complex and transition state [3,4]. Together with the powerful theories, the Arrhenius equation has expanded its application far beyond regular chemical reactions to include diffusion, viscous flow, adsorption-desorption and the denaturation of proteins [5]. Needless to say, the kinetics of phase transitions is a significant application area of the equation [6,7].

In accord with the Arrhenius equation, temperature dependence of the process rate is introduced via the rate constant, *k*:

(1) $$k\equiv k\left(T\right)=A\exp\left(\frac{-E}{RT}\right)$$

where *T* is the absolute temperature, *R* is the gas constant, *A* is the preexponential factor, and *E* is the activation energy. The latter can be defined as follows:

(2) $$E=RT^{2}\frac{d\ln k}{dT}$$

The value of *E* determines the temperature sensitivity of the process rate. The larger the activation energy, the stronger the process rate changes per the same change in temperature. As such, the value of *E* determines another quantity called the temperature coefficient of the rate, *Θ*. This quantity has been introduced by van’ t Hoff and is defined as a change in the rate constant per 10K change in temperature:

(3) $$\ln\Theta \equiv \ln\frac{k\left(T+10\right)}{k\left(T\right)}=\frac{E}{R}\frac{10}{T\left(T+10\right)}$$

*Θ* is associated with the empirical rule formulated by van ‘t Hoff, as “the ratio of velocity constants for two temperatures differing by 10 degrees has a value between 2 and 3 approximately” [8].

Normally, Equation (2) is not employed for experimental evaluations of the activation energy. Such evaluations are commonly made by using another derivative of the rate constant:

(4) $$E=-R\frac{d\ln k}{dT^{-1}}$$

Based on Equation (4), *E* is estimated from the slope of the Arrhenius plot, ln*k* vs. *T*^−1^, which is supposed to be linear (i.e., *E* is supposed to be constant) over the whole temperature range. The principal difference between Equations (2) and (4) is that the former defines the activation energy as a single value averaged over the whole experimental range of temperatures. Equation (2), however, is recommended [9] as a more general definition of the activation energy, because it defines it as a temperature specific value and, thus, permits its evaluation, even if the ln*k* vs. *T*^−1^ plot is not linear. Such behavior is sometimes termed as non-Arrhenian. This terminology is rather confusing, because nonlinear Arrhenius plots can arise from a combination of two or more flawlessly linear ln*k* vs. *T*^−1^ plots, each of which represents perfectly Arrhenian behavior [10].

The occurrence of nonlinear Arrhenius plots has direct relevance to understanding the temperature dependencies of the crystallization and melting rates, as well as to interpreting the experimentally determined activation energies of these processes. One should obviously recognize that a nonlinear Arrhenius plot inevitably yields the activation energy that varies with temperature, i.e., not constant. Preponderance of these phenomena has prompted introduction of the concept of variable activation energy [11,12] that forms a basis to understanding a variety of the condensed phase kinetics [10].

The reliable detection of nonlinear Arrhenius plots requires performing measurements at multiple temperatures (typically no less than 5), spread over a relatively broad temperature range. Due to the technical limitations [13,14], performing such measurements is not a trivial exercise. That is why, nowadays, most of the kinetic measurements on thermally stimulated processes are conducted by means of nonisothermal methods of thermal analysis. For the processes of crystallization and melting, the thermal analysis method of choice is differential scanning calorimetry (DSC).

The heat flow measured by DSC is generally assumed to be directly proportional to the process rate. This assumption is justified by the early work of Borchardt and Daniels [15], who have suggested that one can neglect the thermal inertia term in the heat flow signal. Although neglecting this term introduces some systematic error [16] in the value of the activation energy, it has been demonstrated [17] that this error does not exceed typical experimental uncertainty, provided that one follows the ICTAC recommendations [14] in selecting heating/cooling rates and sample masses. In this circumstance, the process rate measured by DSC can be determined and represented as follows:

(5) $$\frac{d\alpha }{dt}=\frac{1}{Q_{0}}\frac{dQ}{dt}=A\exp\left(\frac{-E}{RT}\right)f\left(\alpha \right)$$

where *α* is the extent of conversion of the reactant to products, *t* is the time, *dQ*/*dt* is the heat flow, *Q*_0_ is the total heat released or absorbed during the process, and *f*(*α*) is the reaction model. A list of the models is available elsewhere [13].

Although nonisothermal measurements can be used for determining the rate constant [13,18,19], it is much easier to use them for directly determining the activation energy. This is accomplished by using the so-called isoconversional derivative of the rate [20].

(6) $$E_{\alpha }=-R{\left[\frac{\partial \ln\left(d\alpha /dt\right)}{\partial T^{-1}}\right]}_{\alpha }$$

where the subscript *α* denotes the values related to a given conversion. This approach gives rise to numerous isoconversional methods and can be applied to both single- and multi-step processes [13,20,21]. All these methods yield the *E_α_* values as a function *α*. For a single-step process, i.e., a process encountering a single energy barrier (cf., Equation (5)), *E_α_* is independent of *α*. A multi-step process encounters more than one energy barrier. For example, two energy barriers (*E*_1_ and *E*_2_) are involved in a process comprising two competing steps. The overall rate of this process is:

(7) $$\frac{d\alpha }{dt}=A_{1}\exp\left(\frac{-E_{1}}{RT}\right)f_{1}\left(\alpha \right)+A_{2}\exp\left(\frac{-E_{2}}{RT}\right)f_{2}\left(\alpha \right)$$

Taking the isoconversional derivative (Equation (6)) of this rate gives *E_α_* that depends on *α* [10]. Such dependence is the equivalent of a nonlinear Arrhenius plot and generally is a sign of a multi-step process.

Nowadays, isoconversional methods represent the most popular way of applying the Arrhenius treatment to kinetic data acquired by means of the thermal analysis techniques. Isoconversional methods have been introduced into the field of polymers in the 1960s [22,23,24,25]. Ever since, their primary application area has been the chemical kinetics of polymers. More recently, the applications of isoconversional methods have expanded into the area of the phase transitions kinetics of polymers [20,26]. As with any novel application, it has been bound to bring about advances and misconceptions. The latter arise primarily from the unwitting transfer of the techniques and interpretations accepted in chemical kinetics into the conceptually different kinetics of phase transitions. The objective of this paper is to provide an overview of the phase transition kinetics with a focus on the temperature dependence of the rates of crystallization and melting as well as on the activation energies of these processes obtained via the Arrhenius kinetic treatment. In this paper, an attempt is made to track the basic ideas to their inception outside the field of polymers. This approach should allow the reader to see the origins of the concepts used and, thus, to get a broader view of the kinetic analyses that utilize them as well as to acquire a deeper understanding of the obtained results.

## 2. Rate of Crystallization

### 2.1. Theoretical Considerations

The formal history of polymers dates back to the seminal paper published by Staudinger in 1920 [27]. This is the same year that Herzog and Jancke employed the X-ray technique to reveal the crystalline nature of the natural polymer, cellulose [28]. A few years later, Staudinger et al. established the crystalline nature of a synthetic polymer, polyoxymethylene [29]. It meant that polymers could crystallize just as low molecular weight compounds.

**Figure 1:**
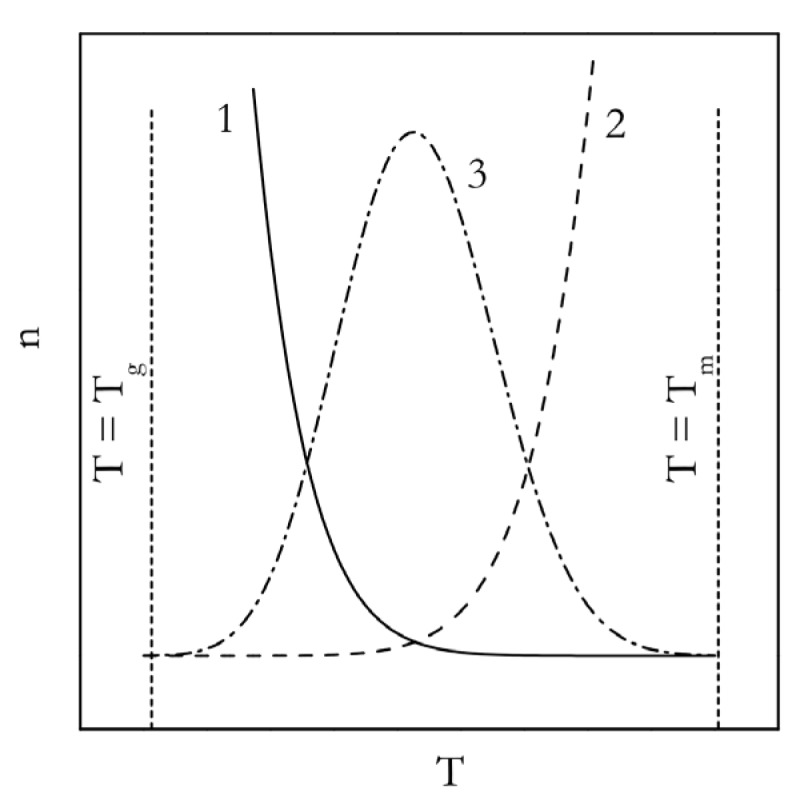
Temperature dependence of the nucleation rate (Equation (8)). 1: exp(−ΔG^*^/RT); 2: exp(−E_D_/RT); 3: product of 1 and 2. Adapted with permission from Vyazovkin [33]. Copyright Elsevier 2008.

Systematic studies of polymer crystallization in a broad temperature range were initiated in 1934 by Bekkedahl [30]. Unfortunately, he did not present the actual dependence of the crystallization rates at different temperatures in that paper. However, he reported that the temperature dependence of the crystallization rate was similar to that known for other organic compounds. That is, crystallization accelerates at small supercoolings, but decelerates at the large ones. The actual bell-shaped dependence of the rate against the set of isothermal temperatures appeared later in a paper by Wood and Bekkedahl [31]. Indeed, this type of dependence had been known for low molecular weight compounds. For example, in 1898, Tammann [32] reported that the temperature dependence of the crystal nucleation rate passes through a maximum for several organic compounds. The shape of this dependence is seen in Figure 1 (curve 3) [33].

This type of temperature dependence is distinctly different from the one observed for the rates of chemical reactions. In accordance with the Arrhenius equation, decreasing temperature causes a chemical reaction to slow down (similar to curve 2 in Figure 1). This is consistent with both activation energy (Equation (2)) and the temperature coefficient (Equation (3)) being positive. However, for crystallization at small supercoolings, i.e., at temperatures moderately lower than the melting temperature, the rate increases with decreasing temperature. This means that the corresponding temperature coefficient and activation energy are both negative. This situation is sometimes referred to as anti-Arrhenian behavior.

Apparently, the origins of the anti-Arrhenian behavior in polymer crystallization were not clear during the early studies. Leo Mandelkern, who started his fundamental work on polymer crystallization as a postdoctoral student under Paul Flory, reminisces about those times (1949–1952): “We found… a strong negative temperature coefficient for the onset of crystallization. An understanding of what was going on completely eluded us for a long time” [34]. Our own experience was also that of a surprise when we discovered large negative values of the activation energy for the crystallization of a polymer melt [35]. Both results have been ultimately explained [35,36] in terms of the nucleation model by Turnbull and Fisher [37]:

(8) $$n=n_{0}\exp\left(\frac{-E_{D}}{RT}\right)\exp\left(\frac{-\Delta G^{*}}{RT}\right)$$

where *n* is the nucleation rate constant, *n*_0_ the preexponential factor, *E_D_* is the activation energy of diffusion, and ΔG^*^ is the free energy barrier to nucleation.

The negative temperature coefficient and/or activation energy for nucleation result from a strong temperature dependence of ΔG^*^. A theoretical treatment of ΔG^*^ is attributed to Volmer [38,39]. It is readily available in a more accessible form from monographs dealing with phase transitions, e.g., [6,7,20,40,41,42], to name a few. For a spherical nucleus, this treatment yields:

(9) $$\Delta G^{*}=\frac{16\pi \sigma^{3}T_{m}^{2}}{3{\left(\Delta H_{m}\right)}^{2}{\left(\Delta T\right)}^{2}}=\frac{\Omega }{{\left(\Delta T\right)}^{2}}$$

where *σ* is the surface energy (surface tension), *T_m_* is the equilibrium melting temperature, Δ*H_m_* is the heat of melting per unit volume, Δ*T* = *T_m_*−*T* is the supercooling, and Ω is a constant that collects all parameters that do not practically depend on temperature. It is clear from Equation (9) that as temperature decreases below *T_m_*, the supercooling becomes larger and the ΔG^*^ value becomes smaller. Since the energy barrier to nucleation becomes smaller, the process rate increases with decreasing temperature (curve 1 in Figure 1). This is the origin of both the negative temperature coefficient and activation energy.

Of course, the above does not explain why at larger supercooling, the crystallization rate starts to decrease with decreasing temperature. This happens because decreasing temperature increases viscosity of the liquid phase and, thus, decreases the diffusion rate of molecules. It means that lowering the temperature slows the rate of self-assembly of the liquid phase molecules into the nuclei of the crystalline phase. This phenomenon is accounted for in Equation (8) by the exponential term containing *E_D_*. The introduction of this term was originally justified by Becker [43]. This term demonstrates the regular Arrhenian behavior, i.e., deceleration with decreasing temperature (curve 2 in Figure 1). Thus, Equation (8) has two exponential terms that respectively demonstrate the Arrhenian (the *E_D_* term) and anti-Arrhenian (the ΔG^*^ term) behavior. The product of these terms gives rise to the temperature dependence of the rate that passes through a maximum (curve 3 in Figure 1) [33]. This explains the bell-shaped temperature dependence of the crystallization rate in polymers as well as in a wide variety of other compounds, including solutions [44,45].

Note that Figure 1 shows the glass transition temperature (*T_g_*) as the lower temperature limit for the nucleation rate. This is not to be understood as being a cessation of nucleation entirely when liquid turns into glass. Active nucleation below *T_g_* has been detected in polymers [46,47] and in lower molecular weight compounds [48,49,50,51]; in the latter case [51], as low as 55 °C below *T_g_*. However, below *T_g_*, the nucleation rate is much slower than in the middle of the *T_g_*–*T_m_* range, where crystallization is typically studied. Therefore, for most practical purposes, *T_g_* can be considered conventionally as the lower temperature limit for the nucleation rate.

**Figure 2:**
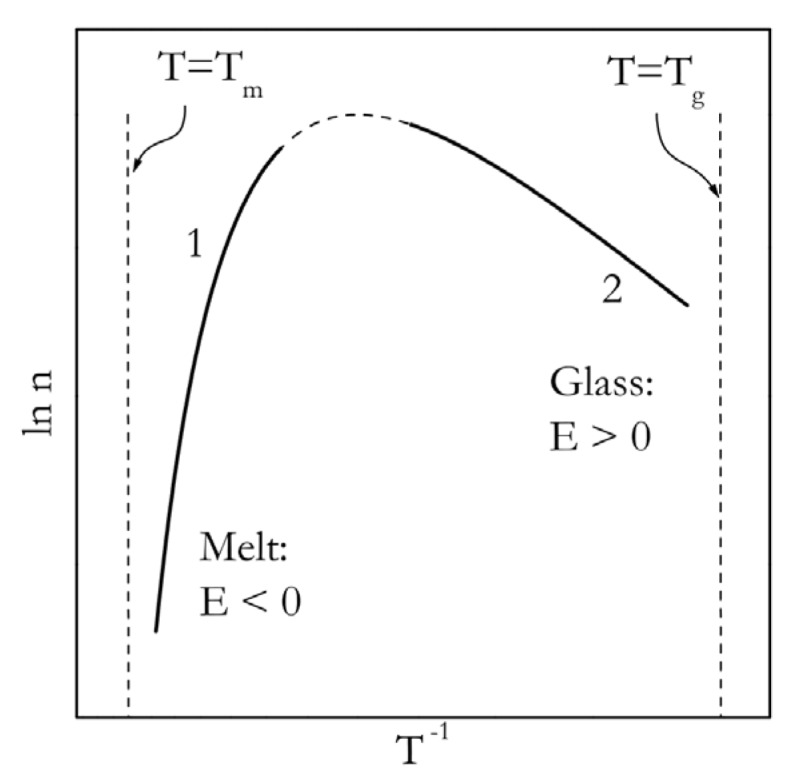
Arrhenius plot for the temperature dependence of the nucleation rate constant (Equation (8)).The bold lines 1 and 2 represent, respectively, crystallization from the melt and glass states. Adapted with permission from Vyazovkin [33]. Copyright Elsevier 2008.

To better understand the Arrhenius activation energies that one can obtain experimentally from the temperature dependencies of the nucleation rate, we need to present Equation (8) in the form of the Arrhenius plot, i.e., as the logarithm of the rate constant against the reciprocal temperature. According to Equation (4), the slope of such plot equals –*E*/*R*. The respective plot for a wide range of temperatures is presented in Figure 2. From a practical point of view, it is important to separate the temperature ranges of small and large supercooling. This is because they are normally accessed in different types of experiments. The temperature range of small supercooling is typically accessed by cooling from the melted state, i.e., from *T_m_* down. The range of large supercooling is reached by heating from the glassy state, i.e., from *T_g_* up. In the range of small supercoolings, the Arrhenius plot (curve 1 in Figure 2) has a positive slope, which corresponds to the negative values of the activation energy. For large supercoolings, it is the other way around, i.e., the slope is negative and the activation energy is positive.

The Turnbull–Fisher model is a general model of the condensed phase nucleation. It does not account for the chain folding mechanism, which is specific to the crystallization of polymers. The temperature dependence of the polymer crystallization rate is described appropriately by the model of Hoffman and Lauritzen [52]. The basic equation of this model is:

(10) $$\Lambda =\Lambda_{0}\exp\left[\frac{-U^{*}}{R\left(T-T_{\infty }\right)}\right]\exp\left(\frac{-K_{g}}{T∆Tf}\right)$$

where Λ is the linear growth rate of spherulites, Λ*_0_* is the preexponential factor, U^*^ is the activation energy of the segmental jump, *f* = 2*T*/(*T_m_* + *T*) is the correction factor, *T_∞_* is a hypothetical temperature associated with the cessation of viscous flow, usually taken 30K below *T_g_*. The parameter *K_g_* is defined as:

(11) $$K_{g}=\frac{lb\sigma \sigma_{e}T_{m}\;}{\Delta h_{f}k_{B}\;}$$

where *b* is the surface nucleus thickness, *σ* is the lateral surface free energy, *σ_e_* is the fold surface free energy, Δ*h_f_* is the volumetric heat of fusion, *k_B_* is the Boltzmann constant, and *l* is a constant that specifies the crystallization regime.

**Figure 3:**
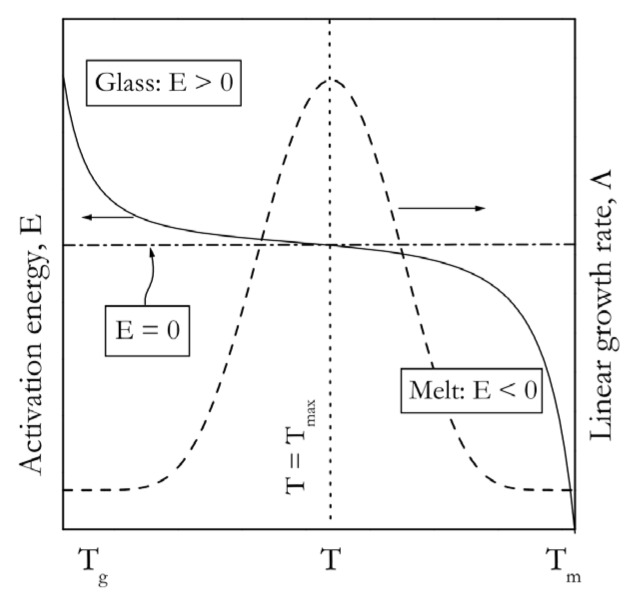
Temperature dependencies of the crystal growth rate (dash line) and activation energy (solid line) that arise respectively from Equations (10) and (12). Adapted with permission from Vyazovkin and Dranca [54]. Copyright 2006 Wiley-VCH.

Mathematically and conceptually, Equation (10) is very similar to Equation (8). The parameters U^*^ and *K_g_* are close analogues of *E_D_* and ΔG^*^. The exponential terms containing U^*^ and *K_g_* respectively have Arrhenian and anti-Arrhenian behavior, so that the product of these terms yields a bell-shaped temperature dependence, as shown in Figure 3. Equation (10) can also be cast in the form of the Arrhenius plot to get an idea about the behavior of the activation energy. Alternatively, one can use Equation (10) to derive the activation energy directly [53]:

(12) $$E=U^{*}\frac{T^{2}}{{\left(T-T_{\infty }\right)}^{2}}+K_{g}R\frac{T_{m}^{2}-T^{2}-T_{m}T}{{\left(T_{m}-T\right)}^{2}T}$$

The resulting activation energy is clearly temperature dependent. This dependence is depicted in Figure 3 [54]. Again, we can see that the *E* values are negative when crystallization is induced by cooling the melt (temperature range *T_max_*–*T_m_*), and positive when it is induced by heating the glass (temperature range *T_g_*–*T_max_*).

### 2.2. Practical Considerations

The aforementioned bell-shaped temperature dependence of the crystallization rate (Figure 1 and Figure 3) is readily detected in isothermal experiments. This dependence is observed not only for the rate itself, but also for parameters related to the rate. For example, parameters inversely proportional to the rate, such as the crystallization induction time (*t_ind_*) and/or the time to reach 50% of crystallization (*t_0.5_*), pass through a minimum [41]. On the other hand, the temperature dependence of the rate constant of crystallization, *Z*(*T*) passes through a maximum, just as the temperature dependence of the rate constant for nucleation (see Figure 2). This is because the temperature dependence of *Z*(*T*) has a form similar to Equation (8). For isothermal crystallization, *Z*(*T*) is typically determined by fitting the relative crystallinity (*α*) vs. time data to the Avrami equation:

(13) log[−ln(1−α)]=logZ(T)+mlogt

where *m* is the Avrami exponent [41] and *Z*(*T*) is commonly called the Avrami rate constant. At a constant temperature, *Z*(*T*) is constant so that Equation (13) is linear with respect to log *t*. In accordance with Equation (5), *α* is readily determined from integration of DSC peaks. Obviously, the slope of the Arrhenius plot of ln*Z*(*T*) vs. *T*^−1^ can be used to determine the activation energy of crystallization. The same applies to the Arrhenius plots of ln *t_ind_* or ln *t_0.5_* vs. *T*^−1^. Either of these plots will be nonlinear, as long as the temperature range of crystallization is sufficiently broad. Additionally, the resulting activation energy will vary with temperature and change its sign as shown in Figure 2.

Under nonisothermal conditions, the process rate depends not only on temperature, but also on the rate of heating or cooling. Using faster cooling rates shifts the melt crystallization curves to lower temperatures. The kinetic curves for glass crystallization shift to higher temperatures by employing faster heating rates. As a result, the bell-shaped temperature dependence of the crystallization rate manifests itself in a more indirect form. For instance, one of the most common techniques for analyzing the nonisothermal crystallization kinetics is that of Ozawa [55]. Its basic equation is as follows:

(14) $$1-\alpha =\exp\left[\frac{-\chi \left(T\right)}{\beta^{m}}\right]$$

where *β* is the cooling or heating rate, and *χ*(*T*) is respectively the cooling or heating function. It should be mentioned that *m* in Equation (14) is sometimes termed as the Ozawa exponent. In fact, as discussed elsewhere [56], this value is meant to be the Avrami exponent.

Equation (14) is applied in the logarithmic form:

(15) log[−ln(1−α)]=logχ(T)−mlogβ 

To make Equation (15) linear with respect to log*β*, log*χ*(*T*) should be kept constant. This is accomplished by means of isothermal cuts through the nonisothermal *α* vs. *T* curves obtained at multiple values of *β*. In other words, one substitutes into Equation (15) the *α* values that correspond to the same temperature at different values of *β*. Then, the intercept of the resulting plot yields log*χ*(*T*). In the Ozawa’s model, *χ*(*T*) is proportional to the crystallization rate [55,57], so it behaves similarly to the isothermal rate of crystallization. Namely, for the melt crystallization, *χ*(*T*) increases with decreasing temperature, and for the glass crystallization it increases when temperature increases [57].

Doubtingly, the Avrami rate constant can be determined from nonisothermal data by using the popular technique proposed by Jeziorny [58]. The technique makes use of Equation (13), in which log*Z*(*T*) is replaced with:

(16) $$\log Z{\left(T\right)}^{*}=\frac{\log Z\left(T\right)}{\beta }$$

One should be warned that the transformation presented by Equation (16) makes no sense, because it violates the basic principle of equating physical values. One can equate only the values that have the same units of measurements. The left-hand side of Equation (16) is dimensionless. The right-hand side has the units of the reciprocal heating (or cooling) rate, i.e., the units of time divided over temperature. That is, the equality set by Equation (16) makes as much sense as, for example, the statement that 1 m = 1 g. Not surprisingly, testing the Jeziorny technique on simulated data demonstrates [57] that it yields entirely erroneous values of the Avrami exponent. It is only prudent to recommend avoiding this technique altogether.

Note that the Avrami model is used broadly in describing the crystallization kinetics of polymers. Yet, it is not necessarily the best way of determining the respective rate constant, *Z*(*T*). First, one should not forget that the model was developed having in mind the crystallization of metals [59]. Thus, it should not be applied uncritically to polymers, whose crystallization is quite different from that of metals [56]. Second, the Avrami model is the only one representative of a broader class of models that describe sigmoid kinetic curves (*α* vs. *t*) [13], such as those typically measured for the isothermal crystallization of polymers and low molecular weight compounds. For example, when testing a broader variety of models, one can find [60] that the Prout–Tompkins model provides a significantly better description of polymer crystallization than the Avrami model. Therefore, a priori assuming that the polymer crystallization kinetics obeys the Avrami model may lead to incorrect determination of the crystallization rate constant if this assumption does not hold.

**Figure 4:**
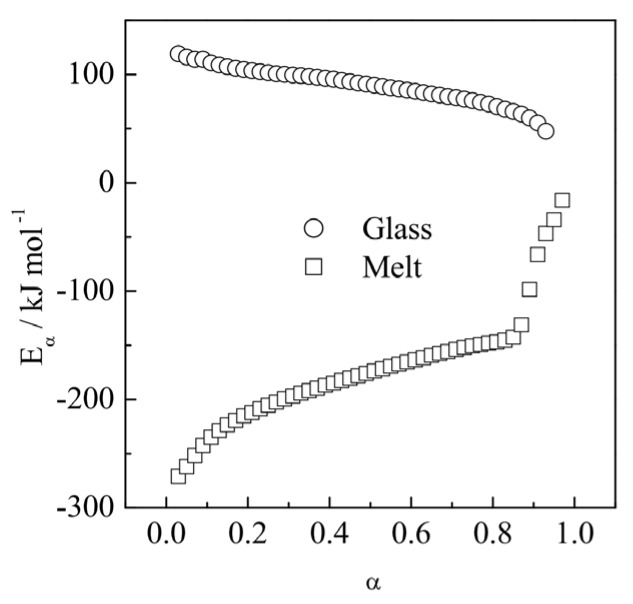
Isoconversional activation energies estimated for crystallization of poly(ethylene terephthalate) by cooling from the melt state ( squares) and by heating from the glass state (circles). Adapted with permission from Vyazovkin et al. [35,54] Copyright 2002, 2006 Wiley-VCH.

As stated in the introduction, it is much easier to use nonisothermal data to obtain the activation energy than the rate constant. For crystallization, the activation energy is readily estimated by an isoconversional method from a series DSC peaks measured at several cooling or heating rates. Generally, the resulting *E_α_* vs. α dependencies should be consistent with the temperature dependencies of the activation energy and rate presented in Figure 3. As an example, we can consider the *E_α_* dependencies estimated for crystallization of poly(ethylene terephthalate) from the melt [35] and glass [54] states (Figure 4). For crystallization from the melt state, *E_α_* demonstrates negative values that increase with increasing *α*. Since the measurements are performed on continuous cooling, an increase in α represents a decrease in *T*. Thus, *E_α_* for crystallization from the melt increases with decreasing temperature. On the other hand, crystallization from the glass state is carried out on continuous heating, so that an increase in α represents an increase in *T*. Thus, the respective decreasing dependence of *E_α_* corresponds to a decrease in *E_α_* with increasing temperature. Therefore, both experimental *E_α_* dependencies presented in Figure 4 are consistent with the theoretical dependence of the activation energy on temperature shown in Figure 3.

The consistency of the experimental dependencies of *E_α_* vs. *T* estimated by an isoconversional method with the theoretical ones (Equation (12)) affords the possibility of estimating the Hoffman–Lauritzen parameters. This is accomplished by fitting Equation (12) to the experimental *E_α_* vs. *T* dependence, which is determined from the dependence of *E_α_* on *α*, by replacing the *α* values with the respective mean temperatures. Details of this technique are described elsewhere [10,53,54].

When it comes to evaluating the activation energy of nonisothermal crystallization, one must be careful in selecting appropriate methods. The major problem here is that many very popular methods cannot be applied to data obtained on cooling, i.e., to crystallization from the melt state. In particular, the highly popular method of Kissinger [61] has been demonstrated [62] to fail in producing correct values of the activation energy from cooling data. As explained at length in previous publications [20,21], the same problem pertains to the so-called rigid integral isoconversional methods, that include Ozawa [23], Flynn-Wall [24,25], and other popular methods. For a proper kinetics analysis of cooling data, one has to use either flexible integral [20,21] or differential isoconversional methods. Among many flexible integral methods, the methods of Vyazovkin [63] and Ortega [64] are used most commonly. The most common differential method is that by Friedman [22].

## 3. Rate of Melting

### 3.1. Theoretical Considerations

To determine the activation energy of a process, one should be able to measure its rate in some range of temperatures. This requirement is at odds with the classical thermodynamic model of melting. According to it, melting occurs at a constant temperature, called the equilibrium melting temperature, *T_m_*. When a crystal is heated at a constant heating rate, its temperature rises until reaching *T_m_*. Once at *T_m_*, all supplied heat converts to the entropy of the forming melt, so that the temperature of the sample does not rise until melting is finished. An implicit assumption of this model is that melting occurs at a much faster rate than the delivery of heat into the sample. Although this model holds in the majority of cases, there are many compounds that melt very slowly. Because of that, they can be superheated; i.e., their temperature can be raised significantly above *T_m_*. Superheating tends to occur in the crystals that produce highly viscous melts [65,66]. This indicates that the diffusional resistance exerted by the melt is likely to be an important factor in the retardation of the melting rate [67]. In any event, as long as a compound is capable of superheating, one can study the temperature dependence of the melting rate and determine the activation energy of the process.

The first detailed study of superheating was carried out by Tammann [68], who demonstrated the phenomenon for glucose and fructose in 1910. In the field of polymers, systematic studies of superheating were initiated by Wunderlich and co-workers. In particular, polyethylene was found to melt quite slowly. Namely, at 1 degree above *T_m_*, complete melting took over 100 h [69]. For the first time, temperature dependent kinetic measurements on superheated polyethylene crystals were conducted by Hellmuth and Wunderlich in 1965 [70]. They noted that the kinetic curves of melting at different temperatures can be superimposed by a linear shift along the log-time axis. This feature is well-known [71] as the time-temperature superposition principle that holds for the relaxation modulus described by the exponential relaxation function:

(17) $$G\left(t\right)=G_{0}\exp\left(\frac{-t}{\tau_{c}}\right)$$

where *G*_0_ is the preexponential factor and *τ_c_* is the characteristic time of the process. Apparently, this fact prompted Hellmuth and Wunderlich to suggest [70] that the kinetics of melting measured as a decrease in the weight fraction of the crystalline phase, *C_w_*, can be represented by Equation (18):

(18) $$C_{w}=\exp\left(\frac{-t}{a}\right)$$

where *a* is a temperature dependent constant. No functional form for the temperature dependence of *a* was proposed [70]. However, by considering the similarity of Equations (17) and (18), one could possibly propose *a* to depend on temperature in the same way as *τ_c_* does, i.e., according to either the Arrhenius or Williams–Landel–Ferry or Vogel–Tammann–Fulcher equation [71].

An extensive study of melting of polyethylene oxide was conducted by Kovacs et al. [72] in 1975. They discovered that the rate of melting depends exponentially on superheating. Based on this, it was concluded that melting “involves an activated process related to the excess free energy of the melt with respect to that of the crystal”. In that paper, one finds an equation for the “temperature coefficient” of the melting rate given as follows:

(19) $$\frac{d\ln r}{dT}\approx \frac{p\Delta H_{m}}{\left(1+n\right)RTT_{m}\left(n\right)}$$

where *p* is the degree of polymerization, Δ*H_m_* is the heat of melting per mole of monomer units, *T_m_*(*n*) melting temperature of *n* times folded crystal. Note that the value presented in Equation (19) is not exactly the temperature coefficient (cf., Equation (3)). Regarding the origins of this equation, Kovacs et al. [72] make a reference to an unpublished paper that does not seem to have ever been published. They also state that this equation is similar to the one proposed by Sanchez et al. [73] for the crystal thickening rate. The equation in question is:

(20) $$r=r_{0}\exp\left(\frac{-\nu \Delta H_{m}\left(T_{m}-T\right)}{RTT_{m}}\right)$$

where *r*_0_ is the preexponential factor, ν is the minimum number of units participating in the cooperative backbone motion through the crystal. Although the right-hand sides of Equations (19) and (20) show some similarities, they are not directly comparable because Equation (19) represents the derivative of the rate, whereas Equation (20) the rate itself. From Equation (20), one can obtain similar derivative as follows:

(21) $$\frac{d\ln r}{dT}=\frac{\nu \Delta H_{m}}{RT^{2}}$$

This derivative is readily converted into the Arrhenius activation energy by utilizing Equation (2). It means that the activation energy in Equation (20) is $\nu \Delta H_{m}$. However, in Equation (19), the activation energy is:

(22) $$E=\frac{p\Delta H_{m}T}{\left(1+n\right)T_{m}\left(n\right)}$$

As per Equation (22), the activation energy of melting should increase with increasing temperature. This result casts doubts on the accuracy of Equation (19), for two reasons. First, an increase in the activation energy should generally slow the process rate. Yet, the rate of melting clearly increases with increasing temperature [70,72]. Second, as discussed later (see Equations (26) and (30), the models of nucleation and growth predict the activation energy of melting to decrease with increasing temperature, and this effect is confirmed experimentally [74]. The limited accuracy of Equation (19) was noted by Kovacs et al. [72], who found that the experimentally determined values of the temperature coefficient exceeded those predicted by Equation (19) by roughly an order of magnitude.

In 1977, Czornyj and Wunderlich [75] conducted kinetic measurements on melting of single crystals of polyethylene and discovered that the rate is linearly proportional to the superheating, Δ*T*=*T*−*T_m_*. They mentioned that the melting of aggregates of polyethylene crystals as well as of other polymers did not follow the linear dependence on Δ*T*. Later, in their study of the melting kinetics of selenium, Wunderlich and Shu [76] found that the temperature dependence of the melting rate depended exponentially on superheating:

(23) $$Rate=L\exp\left(\frac{-M}{T\Delta T}\right)$$

where *L* and *M* are constants. It was suggested that this dependence is consistent with a nucleation model, in which the nuclei are cylindrical shape droplets of the amorphous phase.

Several years later, Maffezzoli et al. [77] proposed the treatment of the temperature dependence of polymer melting directly by the Arrhenius Equation (1). This is an empirical approach that has also been taken by other workers to describe the melting kinetics of polymers [78] and low molecular weight compounds [79]. Although an exponential dependence of the melting rate on temperature is not in question, one should notice an important conceptual difference between the Arrhenius equation and, for example, Equation (23). The former predicts that the process approaches zero rate at 0 K, whereas the latter at *T_m_*. That is, the latter reflects the physical reality of melting, whereas the former does not. For this reason, the origins of an equation that explicitly includes the superheating value (e.g., Equation (23) are more likely to be found in a physical model specific to melting.

A kinetic model specific to polymer melting has been proposed by Toda et al. [80] As some workers before, they have found that the rate of melting of certain polymers (viz., isotactic polypropylene, poly(ethylene terephthalate), and poly(ε-caprolactone)) is exponentially proportional to the superheating. In other words, the temperature dependence of the melting rate is similar to that of the crystallization rate in the vicinity of the melting temperature. Therefore, the nucleation kinetics has been employed as the hypothesis for the development of the model that yields the following value for the size of the free energy barrier [80]:

(24) $$\Delta G^{*}=\frac{\pi l\sigma^{2}T_{m}}{\Delta H_{m}\Delta T}=\frac{M}{\Delta T}$$

where *l* is the thickness of the lamellar crystal, and *M* collects all temperature independent parameters in Equation (24). This form of ΔG^*^ is derived under the assumption of cylindrical nucleus. Step-by-step derivations can be found elsewhere [20]. The substitution of this barrier into the Arrhenius equation yields the rate constant for melting as follows:

(25) $$w=w_{0}\exp\left(\frac{-\Delta G^{*}}{RT}\right)=w_{0}\exp\left(\frac{-M}{RT\Delta T}\right)$$

where *w*_0_ is the preexponential factor. Equation (25) is obviously similar to Equation (23), proposed empirically by Wunderlich and Shu [76]. The substitution of *w* into Equation (4) permits the determination of the Arrhenius activation energy of melting [81]:

(26) $$E=M\left[\frac{1}{\Delta T}+\frac{2T}{{\left(\Delta T\right)}^{2}}\right]$$

As one can see from this equation, the activation energy is inversely proportional to superheating, which means it decreases with increasing temperature.

As an alternative to the nucleation model, one can use a model of nuclei growth [6]. This model has been applied successfully to describe the melting kinetics of low molecular weight compounds. Its application to the polymer melting is yet to be explored. According to this model the nuclei growth rate, *u*, depends on temperature as follows [6]:

(27) $$u=u_{0}\exp\left(\frac{-E_{D}}{RT}\right)\left[1-\exp\left(\frac{\Delta G}{RT}\right)\right]$$

where *u*_0_ is the preexponential factor, and Δ*G* is the difference in the free energy of the final and initial phase. Equation (27) is arrived at as the difference between the rates of the forward and reverse transition. *E_D_* presents the barrier for the forward transition, whereas for the reverse transition the barrier is *E_D_*−Δ*G* (Δ*G* < 0).

It is worth mentioning that both Equation (8) and 27 describe the rate of the new phase formation by a nucleation mechanism. However, Equation (8) assumes that the rate is limited by the formation of the nuclei. Equation (27) assumes that the rate is limited by the growth of the existing nuclei. For this reason, the free energy terms in these equations have an entirely different meaning. In Equation (8), ΔG^*^ is the kinetic barrier and, thus, positive. In Equation (27), Δ*G* is the thermodynamic driving force and, thus, negative.

A dependence of the growth rate on the deviation from *T_m_* is introduced into Equation (27) the same way as in the case of the nucleation model, i.e., through an approximate equality [6,20,41,42]:

(28) $$\Delta G=\Delta H_{m}\left(\frac{T_{m}-T}{T_{m}}\right)$$

Substitution of Equation (28) into (27) yields:

(29) $$u=u_{0}\exp\left(\frac{-E_{D}}{RT}\right)\left[1-\exp\left(\frac{\Delta H_{m}\left(T_{m}-T\right)}{RTT_{m}}\right)\right]$$

Then, by taking the respective derivative (Equation (4)) of *u*, one obtains the activation energy of melting as [74]:

(30) $$E=E_{D}-\frac{\Delta H_{m}\exp\left[\frac{\Delta H_{m}\left(T_{m}-T\right)}{RTT_{m}}\right]}{\exp\left[\frac{\Delta H_{m}\left(T_{m}-T\right)}{RTT_{m}}\right]-1}$$

The subtrahend in Equation (30) is negative and approaches zero with increasing temperature. As a result, the activation energy of melting decreases with increasing temperature approaching asymptotically the activation energy for diffusion.

Note that at small values of the argument, exp(*x*) ≈ *x* + 1. This approximation simplifies Equation (29) to:

(31) $$u=u_{0}^{’}\exp\left(\frac{-E_{D}}{RT}\right)\left(T-T_{m}\right)$$

where $u_{0}^{’}$ contains several temperature independent parameters including Δ*H_m_* and *T_m_*. Such equation has been used by Turnbull et al. [65,82] to describe the melting kinetics of several low molecular weight compounds. It should be stressed that Equation (31) suggests that the rate of melting is an approximately linear function of superheating. Recall that this is the effect reported by Czornyj and Wunderlich [75] for melting of single crystals of polyethylene.

**Figure 5:**
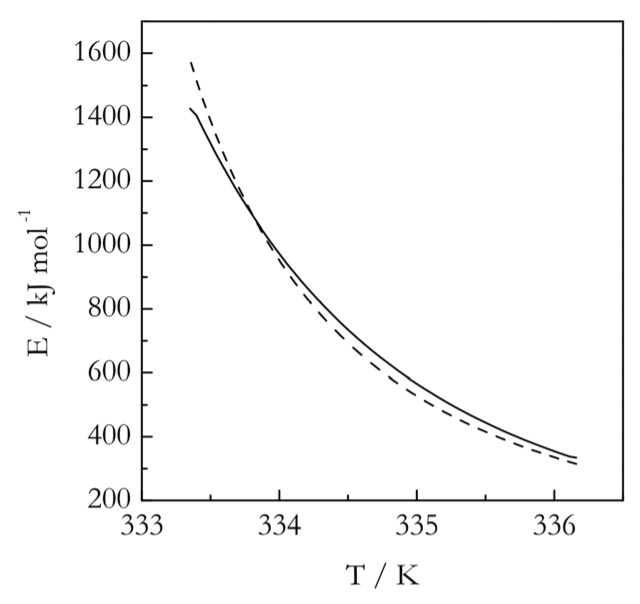
Temperature dependencies of the activation energy for melting of poly(ε-caprolactone) estimated experimentally (solid line). Dashed line represents a fit by Equation (26). Adapted with permission from Vyazovkin et al. [83] Copyright 2014 Wiley-VCH.

An important fact is that either the nucleation model or the model of nuclei growth predicts the activation energy to decrease with increasing temperature. This is the type of dependence that has been observed experimentally for melting of poly(ethylene terephthalate) [81], poly(ε-caprolactone) [83], as well as of glucose and fructose [74]. An example is displayed in Figure 5. In addition, both models indicate that at larger superheatings, the activation energy of melting should approach *E_D_*, i.e., the activation energy of diffusion [67,74]. As already stated, this follows directly from Equation (30). The same result is obtained, if the nucleation Equation (25) is written in a complete form, that includes the diffusion term from the Turnbull–Fisher model (Equation (8)). Then, Equation (26) becomes:

(32) $$E=E_{D}+M\left[\frac{1}{\Delta T}+\frac{2T}{{\left(\Delta T\right)}^{2}}\right]$$

which tends to *E_D_* at large Δ*T*. In the opposite limit of very small superheatings, i.e., at temperatures close to *T_m_*, both Equations (30) and (32) suggest that *E* should take on very large values (see, for example, Figure 5).

**Figure 6:**
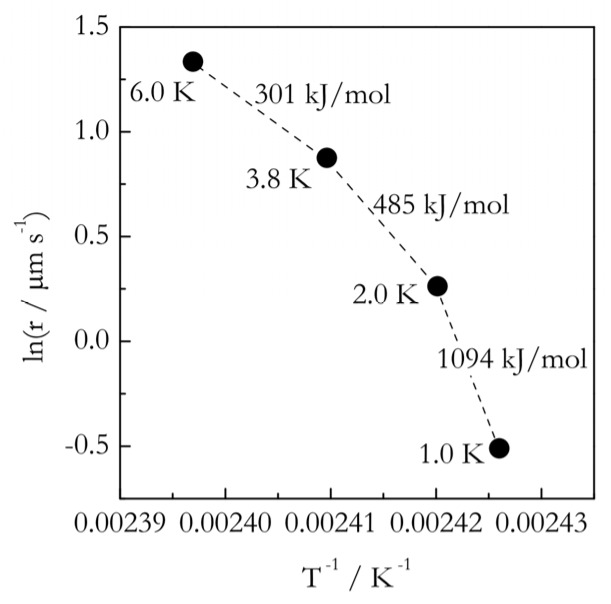
Arrhenius plot for the rate (r) of polyethylene melting (data from Czornyj and Wunderlich [75]). Numbers by the points represent the value of superheating at given temperature. Numbers by the dashed segments are estimates for the corresponding activation energies.

In connection with the afore-discussed temperature dependence of *E*, one can expect the melting kinetics to demonstrate nonlinear Arrhenius plots that are concave down. As an example we can consider the Arrhenius plot (Figure 6), obtained by using the melting rate data for single crystals of polyethylene [75]. As expected, the curvature of the plot decreases with increasing the superheating. For Δ*T* = 1.0–2.0 K, *E* is above 1000 kJ·mol^−1^, whereas at 3.8–6.0 K, it drops down to 300 kJ·mol^−1^. That is, the plot becomes flatter with increasing the superheating and should ultimately yield the slope corresponding to the activation energy of diffusion. Whether this really happens is an open question.

Typically, practically accomplishable superheatings are quite small, i.e., just a few degrees, which is not likely to be enough to reach the temperature range controlled entirely by diffusion. For example, in Figure 6, the activation energy associated with the largest superheating is ~300 kJ·mol^−1^, which is definitely much larger than *E_D_*. The latter is easy to estimate from rheological measurements as the activation energy of viscous flow, *E_η_*. According to the Einstein–Stokes equation:

(33) $$D=\frac{RT}{6\pi N_{A}d\eta }$$

where *D* is the diffusion coefficient, *N_A_* is the Avogadro number, *d* is the molecular diameter, and *η* is the viscosity. Considering that both *D* and *η* can be represented [5] by the Arrhenius equation, one can plug *D* into Equation (4) to obtain:

(34) $$E_{D}=E_{\eta }+RT$$

The second addend in the right-hand side of Equation (34) is normally sufficiently small to be neglected. For different kinds of polyethylene, *E_η_* is around 29 kJ·mol^−1^ [84]. Similarly, in Figure 5, the activation energy of melting of poly (ε-caprolactone) at the largest superheating is ~330 kJ·mol^−1^, whereas *E_η_* is only 35–38 kJ·mol^−1^ [85].

**Figure 7:**
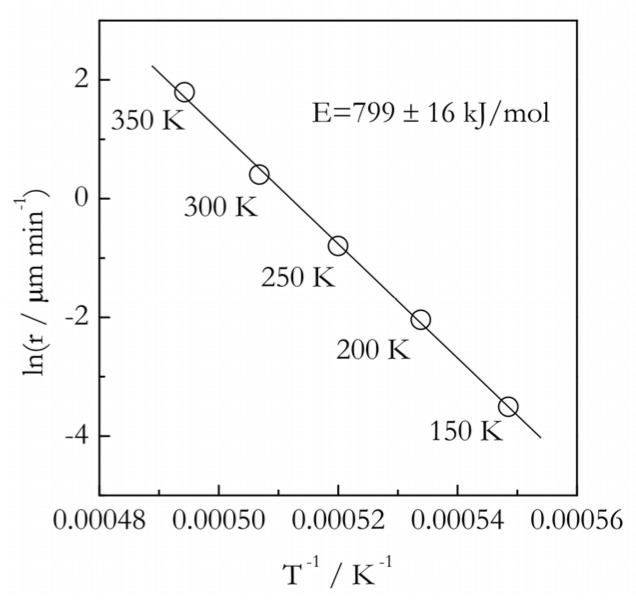
Arrhenius plot for the rate (r) of quartz melting (data from Ainslie et al. [65]). Numbers by the points represent the value of superheating at given temperature. The solid line is a fit to the Arrhenius equation with the activation energy 799 ± 16 kJ·mol^−1^.

Nevertheless, insufficiently large superheatings may not be the only reason as to why the activation energies of melting tend to be markedly larger than those of diffusion or viscous flow. An illuminating example is the melting kinetics of quartz that can be studied at extremely large superheatings. As seen in Figure 7, the respective Arrhenius plot is perfectly linear at the superheatings of 150–350 K. Based on our previous analysis of the nucleation and nuclei growth models, we can expect that, at such large superheatings, the melting kinetics should be controlled by diffusion and should give rise to a linear Arrhenius plot. However, the slope of this plot yields *E* = 799 ± 16 kJ·mol^−1^, whereas *E_η_* for the silica melt is markedly lower, 560 ± 38 kJ·mol^−1^ [86]. Note that, despite a very high temperature (2300 K), the contribution of the *RT* term in Equation (34) is still smaller than the experimental uncertainty. Clearly, even at very large superheatings, i.e., when the melting is presumably controlled by diffusion, the resulting activation energy is still larger than the activation energy of diffusion. On a related note, Liavitskaya et al. [74] have estimated the *E_D_* values by applying the nucleation and nuclei growth models to the melting of glucose and fructose. For both compounds, the *E_D_* values determined by fitting Equation (30) to experimental dependence of *E* vs. *T* have been around 140 kJ·mol^−1^, which is again markedly larger than *E_η_* = 110 kJ·mol^−1^ estimated by rheometry. The *E_D_* values estimated from the nucleation model were even larger.

Of course, at this point, the experimental evidence is too limited to claim that the diffusion barrier that controls melting is necessarily larger than the diffusion barrier generally encountered by molecules in viscous flow. However, this can be justified as a reasonable expectation. As proposed in a theoretical work by Ubbelohde [87], the formation of the liquid phase nuclei occurs via the cooperative rearrangement of molecules on the crystalline surface. This means that mass transfer at the crystal-melt interface is most likely to occur via the motion of clusters of molecules. In its turn, the activation energy of diffusion is known [88,89] to be proportional to the size of diffusing species. As long as such clusters are larger than the molecules or their aggregates present in the regular melt, the activation energy of diffusion in the interface region can be expected to be larger.

### 3.2. Practical Considerations

It is significantly more difficult to study the kinetics of melting than that of crystallization. First, superheating during melting is not nearly as common as supercooling during crystallization. Second, even if superheating is observed, its magnitude is usually much smaller than that of supercooling. This means that the temperature region for studying the melting kinetics is normally quite narrow. Experimentally, the melting kinetics can be studied by both isothermal and nonisothermal DSC.

For isothermal conditions, an important experimental problem is the heat-up period. A selected isothermal temperature for melting is reached via nonisothermal heating of a crystalline compound. If the rate of melting is fast at the selected temperature, a significant fraction of the sample can melt during this heat-up period. Therefore, a significant initial portion of the process can be unmeasurable. This problem is alleviated by lowering the temperature and, thus, the rate. This, however, gives rise to another problem. When the rate of melting is slow at the selected temperature, then in the final stages of melting, the heat flow can drop below the detection limit of DSC, that would make a significant final portion of the process unmeasurable.

The aforementioned problems are not encountered in nonisothermal measurements. This does not mean that the studies of nonisothermal melting kinetics are problem free. According to the ICTAC recommendations [13], performing reliable kinetic analysis requires the simultaneous use of data collected at multiple heating rates. Then, the activation energy and, thus, the temperature dependence of the rate can be evaluated from the shift of the rate peaks (d*α*/d*t* vs. *T*) with the heating rate (see Equation (6)). As widely observed for chemical reactions, the rate peaks (and, thus, the DSC peaks) shift to a higher temperature when using faster heating rates. This effect takes its origin in the reaction kinetics. To attain a given extent of conversion, the reaction needs to spend a certain period of time at a given temperature. To attain the same conversion in shorter time, the temperature has to be higher. When the temperature is raised at a constant heating rate, the faster this rate, the less time the reaction spends at each temperature. As a result, the same conversion is attained at a higher temperature when applying a faster heating rate. This result can be rigorously arrived at by integrating the rate Equation (5) for the conditions of a constant heating rate. Thus, the shifts in DSC curves with the heating rate are used broadly for evaluating the kinetics of chemical reactions. For example, the activation energy is commonly determined from the shift in the position of the DSC peak temperature (*T_p_*), with *β* via the Kissinger equation [61]:

(35) $$E=-R\frac{dln\left(\frac{\beta }{T_{p}^{2}}\right)}{dT_{p}^{-1}}$$

Similarly, the isoconversional methods evaluate the activation energy from the heating rate dependence of either the rate (differential methods) or temperature (integral methods) related to a given conversion.

**Figure 8:**
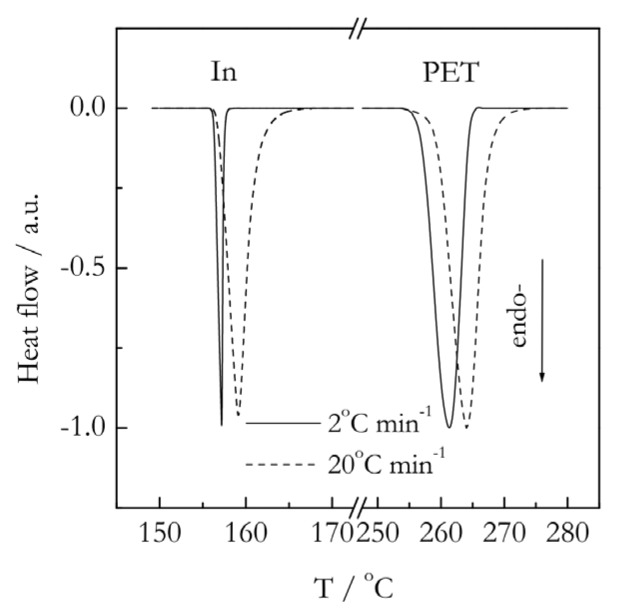
Normalized DSC curves for melting of indium (In) and poly(ethylene terephthalate) (PET) at the heating rates 2 and 20 °C min^−1^. PET data adapted from Vyazovkin [81]. Copyright 2014 Wiley-VCH.

For simplicity, we will use the Kissinger method as an example to clarify why, in the general case of melting (i.e., melting without superheating), one cannot obtain the activation energy of melting from the shift of DSC peaks with temperature. First, we need to recall that during melting, the sample temperature remains constant and independent of the heating rate. The significant shifts of DSC peaks with the heating rate are observed (see Figure 8), because the DSC peaks are normally presented as the heat flow against the reference (furnace) temperature. The latter increases during the entire run, including the temperature region of melting, at a constant rate, *β*. The position of the peak (*T_p_*) is the reference temperature that corresponds to the point when melting is finished, i.e., all crystalline compound has turned into a melt. At a temperature above *T_p_*, the melted sample temperature rises to catch up with the reference temperature. A brief analysis of this situation has been originally done by Illers [90]. More detailed discussions are found elsewhere [91,92].

This analysis suggests that the width of the front part (*T* < *T_p_*) of the DSC melting peak, i.e., the distance between *T_p_* and the equilibrium melting temperature, *T_m_*, is defined as [90]:

(36) $$T_{p}-T_{m}=\sqrt{2R_{sf}\Delta H_{m}m\beta }$$

where *R_sf_* is the thermal resistance to the heat flow between sample and furnace and *m* is the mass. Note that the equilibrium melting temperature, *T_m_* is generally estimated from the DSC melting peak as the extrapolated onset temperature, *T_on_*, so that *T_m_* = *T_on_*. The back part (*T* > *T_p_*) of the DSC melting peak represents the heat flow caused by the melt temperature rising toward the reference temperature. The respective heat flow obeys the exponential relaxation [90]:

(37) $$\frac{dQ}{dt}={\left(\frac{dQ}{dt}\right)}_{p}\exp\left(-\frac{t}{\tau }\right)$$

where the subscript *p* denotes the heat flow value at the peak temperature, and the time constant *τ* is independent of *β* and defined as:

(38) $$\tau =C_{s}R_{sf}$$

where *C_s_* is the total heat capacity of the sample.

All things considered, the thermophysical analysis [90,91,92] of the DSC melting peak suggests that both front and back parts of the DSC melting peak are defined by parameters that do not include either the activation energy or any other kinetic parameters of melting. Additionally, only the front part of the DSC peak represents the melting transition. More importantly, the dependence of *T_p_* on *β* (Equation (36)) is not determined by *E*, as expected from the Kissinger Equation (35). It is determined by the values of *R_sf_*, Δ*H_m_* and *m* (Equation (36)). That is, neither any part of the DSC meting peak or its shift with the heating rate can be used to obtain either the activation energy or any other kinetic parameter of melting. It should be stressed that the difference *T_p_*–*T_on_* (i.e., an analog of the *T_p_*–*T_m_* difference in Equation (36)) can be readily derived for any chemical reaction by integrating Equation (5) from *T_on_* to *T_p_* and applying the mean value theorem. In that case, this difference would be determined exclusively by the kinetic parameters of the reaction.

As already stated, the above analysis applies to the common case of melting without superheating. When a compound melts with superheating, the situation changes drastically and becomes similar to that for chemical reactions. Just as in the case of chemical reactions, the sample temperature changes during melting. Also, the crystalline compound continues to melt in the whole temperature range of the DSC melting peak [70]. As a result, the heat flow generated by such melting becomes determined by the melting kinetics. This justifies one in analyzing the temperature dependence of the melting rate in the frameworks of the Arrhenius equation, including the usage of the Kissinger and isoconversional methods [74,81,83].

As discussed so far, the possibility of using DSC for evaluating the melting kinetics is contingent upon whether a compound undergoes superheating during melting. There are some relatively simple tests that can verify the presence or absence of superheating. Naturally, on might assume that plotting the DSC signal against the sample rather than reference temperature should help to differentiate between the two cases. Indeed, as long as the sample temperature remains constant, the heat flow could be expected to rise strictly perpendicular to the temperature axis. In reality, it is not so, for two reasons. First, per the Gibbs phase rule, for isobaric conditions:

(39) F=C−P+1

(*F*, *C*, and *P* are the numbers of degrees of freedom, components, and phases), the melting temperature for the crystal-melt equilibrium (i.e., *P* = 2) is defined uniquely (i.e., *F* = 0) only for a single component (i.e., *C* = 1) system. In its turn, the condition *C* = 1 holds only for a compound that contains no impurities. In the presence of an impurity, *C* = 2 that makes the melting temperature dependent on the mole fraction of the impurity in the melted phase. This fraction decreases during melting, causing the melting temperature to rise [93]. This effect is used broadly for purity analysis by DSC. The second reason for rising the sample temperature in DSC measurements is rather technical. DSC measures the sample temperature by using the sensor that is in contact with the pan that holds the melting compound. This creates a thermal resistance to the heat flow between sample and sensor, *R_ss_*. Then, the heat flow rises almost linearly under the angle whose tangent is 1/*R_ss_* [94]. Therefore, even for the melting of a highly pure (i.e., single component) compound the strictly vertical rise of the heat flow is impossible, because the value of *R_ss_* is necessarily larger than zero.

More effective tests on the presence or absence of superheating have been proposed by Toda [92]. Two of them include checking the effect of the heating rate and sample mass on the width of the DSC melting peak. In the absence of superheating, the peak width is determined by Equations (36) (for *T* < *T_p_*) and (37) (for *T* > *T_p_*). As per Equation (36), the temperature range *T_p_*–*T_m_* becomes broader in proportion to the square root of the sample mass, as well as of the heating rate. In Equation (37), *τ* is independent of the heating rate, but increases with the mass. Thus, the width of the back part of the peak increases with the mass, but also with the heating rate, because the temperature range is proportional to *βτ*. The broadening is especially easy to see in the normalized DSC peaks shown in Figure 8. It is seen that for melting of indium that occurs without superheating, the DSC peaks become much broader with an increase of the heating rate. On the other hand, the melting of poly(ethylene terephthalate) occurs with superheating, and the respective melting peaks do not reveal any significant broadening, because in this case, the heat flow measured by DSC is controlled by the kinetics of melting. An increase in the sample mass demonstrates similar effects.

Although these two tests are simple and demonstrative, they are not quantitative. A proper quantitative test is based on evaluating the power exponent *z* in [92]:

(40) $$T_{p}=T_{m}+B\beta^{z}$$

where *B* and *z* are fit parameters. It is seen from Equation (36) that in the absence of superheating the DSC peak temperature should shift as a function of the heating rate with *z* = 0.5 as shown by Illers [90]. A recent theoretical analysis by Toda [92] suggests that *z* can take on values from 0.5 to 1. More importantly, it indicates that estimating *z* significantly less than 0.5 can be taken as evidence of superheating. The analysis also demonstrates that for this test to work efficiently, one needs to use the *T_p_* values, not from the raw DSC peaks, but from the peaks adjusted for the thermal inertia term. The adjustment can be performed as follows:

(41) $$\frac{dQ}{dt}=RHF+\tau \frac{d\left(RHF\right)}{dt}$$

where RHF is the raw heat flow, as measured by DSC. The value of the time constant *τ* is the same as in Equation (37). It is estimated from analysis of the back part (*T* > *T_p_*) of a DSC peak measured for melting of a pure metal, e.g., In, as described elsewhere [94]. Care should be taken to use such mass of In that the total heat capacity of the In sample is similar to the total heat capacity of the polymer sample [17]. The total heat capacity involves both the sample and sample holder, e.g., Al pan. Note that the adjustment for thermal inertia (second addend in Equation (41)) causes the *T_p_* value to shift to a lower temperature. The size of this shift increases with the heating rate.

In conclusion, we need to note that Equation (40) follows from the rate equation [80]:

(42) $$\frac{d\alpha }{d\Delta t}=a{\left(\beta \Delta t\right)}^{y}\left(1-\alpha \right)$$

where *α* is the extent of crystal to melt conversion, Δ*t* is a time period, *a* and *y* are parameters. The parameter *y* is related to z as follows:

(43) $$z=\frac{1}{1+y}$$

Clearly, Equation (42) suggests that the rate obeys the power law dependence on temperature. Nevertheless, the Arrhenius treatment discussed in this paper is based on the exponential temperature dependence (Equation (5)). As shown by Vyazovkin [67], these treatments are not mutually exclusive and can be linked to each other. Namely, one can establish a functional dependence between the Arrhenius activation energy and the power law exponent. For example, within the formalism of the Kissinger method, *E* is linked to *z* as:

(44) $$E=\frac{RT_{p}^{2}}{z\left(T_{p}-T_{m}\right)}-2RT_{p}$$

**Figure 9:**
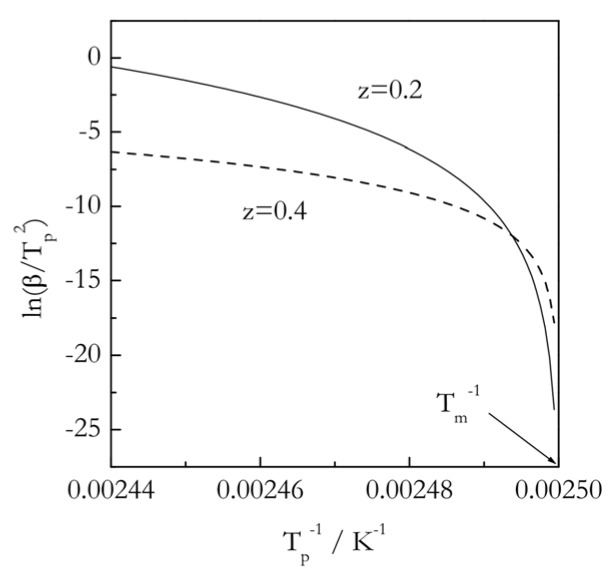
Kissinger plots for a melting process with *T_m_* = 400 K and superheating up to 10 K, for the *z* values 0.2 and 0.4. Adapted from Vyazovkin [67]. Copyright 2018 ACS.

Therefore, knowing the value of *z* from fitting *T_p_* vs. *β* data allows one to estimate the value of *E*. Similarly, one can estimate *z* from the value of *E*. It is noteworthy that smaller values of *z* give rise to the Kissinger plots of larger curvature, as illustrated in Figure 9. The larger the curvature, the stronger the dependence of the activation energy on temperature. It is also seen that the activation energy increases dramatically when temperature is decreased to closely approach *T_m_*. A similar effect is observed in the Arrhenius plot for isothermal data (Figure 6). The effect is consistent with the predictions of Equations (30) and (32) for very small superheatings.

Similar behavior of the activation energy is observed around the equilibrium temperature for other reversible processes taking place on heating, such as gelation [95], coil-to-globule [96] and solid-solid [97] transitions as well as reversible thermal decompositions [98]. For all these processes, the rate is determined not only by the activation energy of the forward step, but also by the magnitude of the departure from equilibrium, i.e., by the thermodynamic driving force. The latter, being temperature dependent, makes an additional contribution to the temperature dependence of the rate and, therefore, to the activation energy measured experimentally from such dependence. Generally, the temperature sensitivity of the rate is inversely proportional to the thermodynamic driving force. For this reason, the temperature sensitivity is the largest when the process is the closest to equilibrium, so that one unavoidably finds very large values of the experimental activation energy when the temperature range of measurements approaches the equilibrium temperature.

## 4. Conclusions

The present review demonstrates that the processes of crystallization and melting are kinetically complex. It means that the temperature dependence of these processes is determined by more than one energy barrier and/or energy barriers, whose size depends on temperature. From standpoint of the Arrhenius treatment it means that one should generally expect the respective Arrhenius plots to be nonlinear and, therefore, the activation energy to vary with temperature. The considered models afford an understanding of the origins of the experimentally observed nonlinear plots and variations in the Arrhenius activation energy. Furthermore, fitting the theoretical temperature dependencies to the experimental ones may permit the evaluation of important intrinsic parameters of the process.

The science of polymers started to develop years later than that of lower molecular weight compounds. Naturally, many fundamental concepts created while studying simpler inorganic and organic compounds have been introduced in the field of polymers. This approach has proved to be fruitful, as long as one takes into account the behavior specific to polymers. A good example here is the Hoffman–Lauritzen model that adjusted the Turnbull–Fisher formalism to the crystallization of polymers. Compared to the crystallization kinetics, the kinetics of melting remains largely underdeveloped, primarily due to the significant experimental difficulties associated with the respective studies. For example, the nucleation model of melting has so far received only limited testing. At the same time, the nuclei growth model, frequently applied to the melting kinetics of low molecular weight compounds, is yet to be explored for the melting of polymers.

Overall, it is hoped that the theoretical considerations offered in this review will be helpful in understanding and interpreting the results of the Arrhenius treatment (including the treatment by isoconversional methods) of the data on crystallization and melting of polymers. On the other hand, the presented practical considerations are expected to help in identifying proper computational methods and experimental data that can lead to meaningful kinetic interpretation.
